# Clinical feasibility of ultrafast intracranial vessel imaging with non-Cartesian spiral 3D time-of-flight MR angiography at 1.5T: An intra-individual comparison study

**DOI:** 10.1371/journal.pone.0232372

**Published:** 2020-04-29

**Authors:** Thomas Sartoretti, Elisabeth Sartoretti, Árpád Schwenk, Luuk van Smoorenburg, Manoj Mannil, André Euler, Anton S. Becker, Alex Alfieri, Arash Najafi, Christoph A. Binkert, Michael Wyss, Sabine Sartoretti-Schefer

**Affiliations:** 1 Institute of Radiology, Winterthur Cantonal Hospital, Winterthur, Switzerland; 2 Institute of Diagnostic and Interventional Radiology, University Hospital Zürich, University of Zürich, Zürich, Switzerland; 3 Department of Neurosurgery, Winterthur Cantonal Hospital, Winterthur, Switzerland; 4 Philips Healthsystems, Zürich, Switzerland; Medical University of Vienna, AUSTRIA

## Abstract

**Objectives:**

Non-Cartesian Spiral readout can be implemented in 3D Time-of-flight (TOF) MR angiography (MRA) with short acquisition times. In this intra-individual comparison study we evaluated the clinical feasibility of Spiral TOF MRA in comparison with compressed sensing accelerated TOF MRA at 1.5T for intracranial vessel imaging as it has yet to be determined.

**Materials and methods:**

Forty-four consecutive patients with suspected intracranial vascular disease were imaged with two Spiral 3D TOFs (Spiral, 0.82x0.82x1.2 mm^3^, 01:32 min; Spiral 0.8, 0.8x0.8x0.8 mm^3,^ 02:12 min) and a Compressed SENSE accelerated 3D TOF (CS 3.5, 0.82x0.82x1.2 mm^3^, 03:06 min) at 1.5T. Two neuroradiologists assessed qualitative (visualization of central and peripheral vessels) and quantitative image quality (Contrast Ratio, CR) and performed lesion and variation assessment for all three TOFs in each patient. After the rating process, the readers were questioned and representative cases were reinspected in a non-blinded fashion. For statistical analysis, the Friedman and Nemenyi post-hoc test, Kendall W tests, repeated measure ANOVA and weighted Cohen's Kappa tests were used.

**Results:**

The Spiral and Spiral 0.8 outperformed the CS 3.5 in terms of peripheral image quality (p<0.001) and performed equally well in terms of central image quality (p>0.05). The readers noted slight differences in the appearance of maximum intensity projection images. A good to high degree of interstudy agreement between the three TOFs was observed for lesion and variation assessment (*W* = 0.638, p<0.001 –*W* = 1, p<0.001). CR values did not differ significantly between the three TOFs (p = 0.534). Interreader agreement ranged from good (*K* = 0.638) to excellent (*K* = 1).

**Conclusions:**

Compared to the CS 3.5, both the Spiral and Spiral 0.8 exhibited comparable or better image quality and comparable diagnostic performance at much shorter acquisition times.

## Introduction

Time-of-Flight (TOF) Magnetic Resonance Angiography (MRA) is a non-invasive and contrast-media free MR technique that has been widely adopted in clinical institutions for intracranial vessel imaging in patients with suspected intracranial arterial disorders (stenosis, aneurysms etc.) [1–8]. TOF MRA has been shown to be a viable alternative to Digital Subtraction Angiography (DSA) [1–8]. However, conventional TOF-MRA imaging can be time consuming, especially when performing image acquisitions with sub-millimeter resolution [9].

To this extent, image acquisition acceleration techniques have been developed. Parallel imaging (PI) [10–12] is considered a common k-space undersampling method that relies on the reduction of the number of phase-encoding steps. The acceleration factor chosen for PI imaging however rarely exceeds 2 or 3 and thus PI accelerated TOFs are still quite time consuming, thereby, depending on the spatial resolution, rarely achieving scan times below 5 minutes [2]. A further reduction of acquisition times is achievable with compressed sensing technology (CS). CS algorithms achieve higher k-space undersampling by using the underlying sparsity in an appropriate transform domain [1–9].

Several recent studies have clearly demonstrated the clinical feasibility and efficacy of CS accelerated TOFs, which can therefore be considered a modern reference standard for clinical grade TOF imaging [1–9]. Of the various sequences used in these studies, the fastest ones achieved scan times of slightly above 2:30 minutes [1,2,6,7]. Against this background, while desirable [9, 13], a further reduction of acquisition times considerably below 2 min seems improbable with the currently employed acceleration techniques [8].

However, spiral MR imaging with a non-Cartesian acquisition technique potentially enables higher scan speeds, as the spiral traverses the k-space more efficiently per given time than in Cartesian trajectories [14–18].

Therefore, novel prototypical TOF MRA sequences with spiral readouts have been developed recently. While these Spiral-TOFs have shown promising results in a volunteer study at 1.5T [8] and in a recent clinical pilot study at 3T [9], the evidence for their applicability in a clinical setting is scarce. Thus further validation of their clinical utility, especially at a field strength of 1.5T, is required before clinical implementation.

Therefore, in this intra-individual comparison study, we assessed the clinical feasibility of Spiral-TOFs for intracranial vessel imaging at 1.5T by comparing two Spiral-TOFs with a CS accelerated TOF.

## Materials and methods

### Study design

In this intra-individual comparison study, we assessed the clinical feasibility of Spiral-TOFs for intracranial vessel imaging at 1.5T by comparing two Spiral-TOFs with a CS accelerated TOF by means of qualitative and quantitative image quality metrics and lesion and variation assessment as a measure for diagnostic efficacy.

### Ethics statement

This study was approved by the Cantonal Ethical Committee Zurich, Zurich, Switzerland (BASEC Number 2018–01275) with general written informed consent from all patients.

### Patient selection

From April to June 2019, 44 consecutive patients (19 male, 25 female, age: 64 ± 17) were enrolled in this intra-individual comparison study. The prototypical Spiral-TOFs were added to the routine MR protocol for patients evaluated for suspected intracranial vascular disease (i.e. stenoses, aneurysms, intracranial hemorrhage, vasculitis, reversible vasoconstriction syndrome). All patients had been referred to our radiology department by neurologists and physicians of our hospital. All patients who successfully completed the whole MR examination were included in the study. All 44 patients completed the MR examination and thus no patient had to be excluded.

### MR imaging

All patients were scanned with a 1.5T scanner (Ingenia, Philips Healthcare, Best, the Netherlands) using a 16-channel head coil and product software (R5.6). In addition to the three TOF sequences, the following sequences were acquired as part of the comprehensive clinical routine MR examination,: transverse diffusion weighted echo planar MRI (DWI), sagittal 3D fluid attenuated inversion recovery (FLAIR), transverse 3D susceptibility weighted imaging (SWI), sagittal 3D T1 turbo field echo (TFE) (known as MPRAGE), transverse T2 weighted turbo spin echo (TSE).

**Time-of-flight MRA imaging.** The TOF sequences used in this study are identical to those evaluated and validated in a previous, preclinical study [8] and represent 3D TOF MRA sequences. The sagittal 3D T1w TFE sequence (Field of view 240 x 240 cm2, matrix 240 x 240, nr. of slices 275, TR / TE / TI 7.6 ms / 3.5 ms / 1000 ms, flip angle 8°, scan duration 03 min 50 sec) was used for planning of the TOFs. The three TOF sequences were acquired in identical orientation parallel to the splenium and genu of the corpus callosum. All TOFs were based on a multi-chunk (5 chunks) acquisition and a TONE ramp of 17° at the entry slice, 20° at mid-slab, and 23° near the exiting slice.

Two sequences had an identical geometric resolution (voxel size: 0.82 x 0.82 x 1.2 mm^3^); First the Cartesian CS 3.5 sequence (used routinely at our and other institutions [9]) relying on the Compressed SENSE acceleration technique with a variable density Poisson disk-sampling scheme followed by iterative reconstruction (Compressed SENSE factor 3.5, scan duration 03:06 min). Details concerning the Compressed SENSE technique can be found elsewhere [13,19,20,21,22,23]. Second a prototypical non-Cartesian Spiral-TOF (abbreviated Spiral) based on a stack of spirals with an in-plane spiral-out readout scheme (spiral interleaves 27, spiral acquisition window 10 ms, scan duration 01:32 min). In addition, a further prototypical non-Cartesian Spiral-TOF with the same spiral-out readout scheme but higher resolution was acquired: The isotropic Spiral-TOF 0.8 (abbreviated Spiral 0.8), (voxel size 0.80 x 0.80 x 0.80 mm^3^, scan duration 02:12 min). Blurring due to off-resonance was corrected during reconstruction based on a magnetic field map acquired prior to the spiral scan (included in the acquisition time shown in **Table 1**). The sequences' parameters were defined based on the vendor's implementation. The isotropic Spiral 0.8 sequence was added to the protocol, as it was described to be the most promising candidate for clinical use of all Spiral-TOF sequences tested in a previous preclinical study [8].

**Table 1:** Sequence parameters of the TOF MRA sequences.

	CS 3.5	Spiral	Spiral 0.8
Field of view [mm]	200 x 200 x 90	200 x 200 x 90	200 x 200 x 90
Acq. voxel size [mm]	0.82 x 0.82 x 1.2	0.82 x 0.82 x 1.2	0.82 x 0.82 x 0.82
Rec. voxel size [mm]	0.39 x 0.39 x 0.6	0.39 x 0.39 x 0.6	0.39 x 0.39 x 0.41
Nr. of slices	150	150	220
TR / TE [ms]	23 / 6.9	23 / 2.3	23 / 2.3
Flip angle [degree]	20	20	20
SENSE factor	n.a.	n.a.	n.a.
C-SENSE factor	3.5	n.a.	n.a.
Spiral acq. window / Nr. of spiral interleaves	n.a.	10 ms / 27	10 ms / 27
Saturation Slab	1	1	1
Receiver bandwith	108 Hz / pixel	100 Hz / pixel	100 Hz / pixel
Nr. of signal averages	1	1	1
Flow compensation	Yes	Yes	Yes
TONE ramp pulse	Yes	Yes	Yes
Nr. of chunks	5	5	5
Scan duration [min:sec]	03:06	01:32	02:12

Details of the imaging parameters of the three TOFs are given in **Table 1**. A further description of the TOFs, including a diagram of the spiral acquisition can be found elsewhere [8].

### Qualitative image quality

Two readers (S.S, board certified neuroradiologist with 30 years of post-fellowship experience and A.S, board certified neuroradiologist with 7 years of post-fellowship experience) independently evaluated maximum intensity projections (MIP, rotation in both directions and generated without further post processing from source images) and source images [1,4,7] and were thereby blinded to the patient details and imaging technique [1]. All images were transferred to the IntelliSpace portal workstation (Version 10, Philips Healthcare, Best, the Netherlands) and displayed to the readers in a random order and with image annotations switched off (blinded read out). Specifically, the images from a single patient did not necessarily appear in sequential order. The readers were then asked to rate the qualitative image quality with a 5-point Likert-type scale for all TOF images of each patient. Specifically, they were asked to separately rate the central image quality encompassing the following structures: (Intracranial carotid arteries with intraosseous, cavernous and intradural segments, vertebral arteries V4 segments, basilar artery, posterior cerebral arteries P1 segments, middle cerebral arteries M1 segments and anterior cerebral arteries A1 segments) and the peripheral image quality encompassing the following structures: (posterior cerebral artery P2 to P4 segments, middle cerebral artery segments M2 to M4 segments, anterior cerebral artery segments A2 to A4 segments).

The 5-point Likert-type Scale was adapted from Lin et al. [1] and was defined as follows: 1) Nondiagnostic image quality caused by severe artifacts, poor signal intensity and image distortions. 2) Poor diagnostic image quality thus resulting in poor diagnostic confidence of the readers caused by image distortions, severe artifacts and poor signal intensity of vessels. 3) Moderate image quality and thus moderate confidence of the readers because of moderate artifacts and moderate image distortions. 4) Good image quality with well-delineated vessels and only slight artifacts and image distortions. 5) Excellent image quality with no artifacts or image distortions and flawless visualization of vessels.

After the readout, the readers were questioned about their rating experience and 10 relevant cases were reinspected in a non-blinded fashion.

### Lesion and variation assessment

As a measure for diagnostic efficacy, lesion (aneurysm, stenosis) and variation (congenital absence, hypoplasia and variant branching) assessment was conducted as proposed by Lin et al. [1]. The assessment was performed by two readers (S.S and A.S) on source images and MIPs in a blinded and randomized manner on the following vessel segments: Intracranial carotid arteries with intraosseous, cavernous and intradural segments, vertebral arteries V3-V4 segments, basilar artery, P1-P3 segments of the posterior cerebral arteries, M1-M3 segments of the middle cerebral arteries, A1-A3 segments of the anterior cerebral arteries, anterior communicating artery (AcoA) and bilateral posterior communicating arteries (PcoAs). For both AcoA and bilateral PcoAs only the absence and presence of aneurysms were evaluated.

Readers were asked to record all lesions and variations for the arterial segments described above. Concerning arterial stenoses, the readers were asked to apply a 5-point semi-quantitative grading of the degree of stenoses: 1) nonstenosis, stenosis = 0%; 2) mild, stenosis ≦50%; 3) moderate, 50% < stenosis <70%; 4) severe, 70% ≦ stenosis <100%; 5) occlusion, stenosis = 100%. [1].

### Quantitative image quality—contrast ratio (CR)

As a quantitative measure for image quality, contrast ratio (CR) measurements were obtained for all three TOFs with the following formula [7]:

$$CR=\frac{\left(SI_{Vessel}-\;SI_{Background}\right)}{\left(SI_{Vessel}+\;SI_{Background}\right)}$$

SI_Vessel_ and SI_Background_ represent signal intensities (SI) as derived from region of interest (ROI) measurements on source images [1,24]. For SI_Vessel_, ROIs were placed on the proximal left M1 segment of the middle cerebral artery while for SI_Background_,ROIs were placed on the adjacent gray matter. In case of stenosis or occlusion of the left middle cerebral artery, the right middle cerebral artery was selected for ROI measurements [1]. Slight anatomical variations were taken into account by slightly varying the size of ROIs [7]. ROI measurements were performed by one reader (S.S) and checked by a second reader (A.S) [1,19,24].

### Statistical analysis

Except for CR, values are reported separately for reader 1 and 2. Histograms, Q-Q Plots and Shapiro Wilks tests were used to check the distribution/normality of data. To compare qualitative image quality (central and peripheral) between the three TOFs, the Friedman test and Nemenyi post-hoc test for pairwise comparisons was used. To assess the interstudy agreement between the three TOFs for lesion and variation assessment, Kendall W tests were applied (0.50 < W ≦ 0.80, good agreement; 0.80 < W, excellent agreement) [1,24]. P-values from the Kendall W tests were corrected with the Bonferroni-Holms method. To compare CR values between the three TOFs, repeated measure ANOVA was utilized. To assess interreader agreement, weighted Cohen's Kappa analyses were performed (kappa ≦ 0.40, poor agreement; 0.40 < kappa ≦ 0.60, moderate agreement; 0.60 < kappa ≦ 0.80, good agreement; and kappa > 0.80, excellent agreement). Unless indicated otherwise, values are reported as (mean ± standard deviation (SD)). A p-value < 0.05 was considered significant. All analyses were performed in the R programming language (version 3.6.1) (R Core Team, 2019). The package “irr” (Gamer, Lemon, Fellows, Singh, 2012) was used to compute weighted Cohen's Kappas. The package «PMCMR» (Pohlert, 2018) was used to calculate the Friedman and Nemenyi post-hoc tests and the package "ggplot2" (Wickham et al. 2020) was used to compute boxplots.

## Results

### Reduction of acquisition times

Concerning the reduction of acquisition times, the Spiral (01:32 min) achieved a reduction of 50.54% while the Spiral 0.8 (02:12 min) achieved a reduction of 29.03% in comparison to the CS 3.5 sequence (03:06 min).

### Qualitative image quality

**Fig 1:**
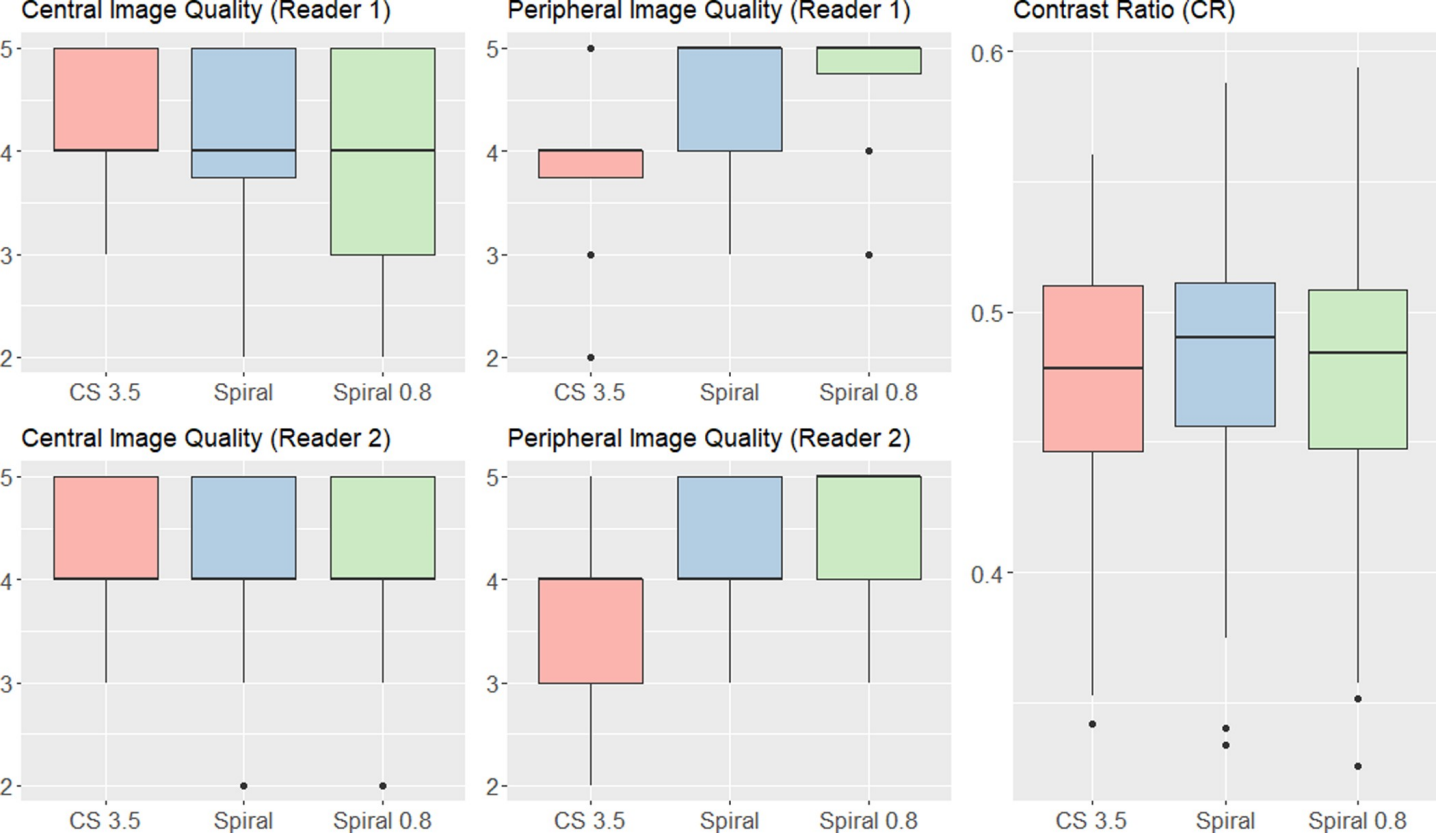
Visualization of the data from qualitative and quantitative image quality assessment. The data is depicted with boxplots. The prominent black lines within the boxplots represent the medians. The hinges of the boxplots mark the 25^th^ and 75^th^ percentiles. The upper/ lower whiskers extend from the hinges to the largest/smallest value no further than 1.5 * Interquartile Range from the hinge.

**Fig 2:**
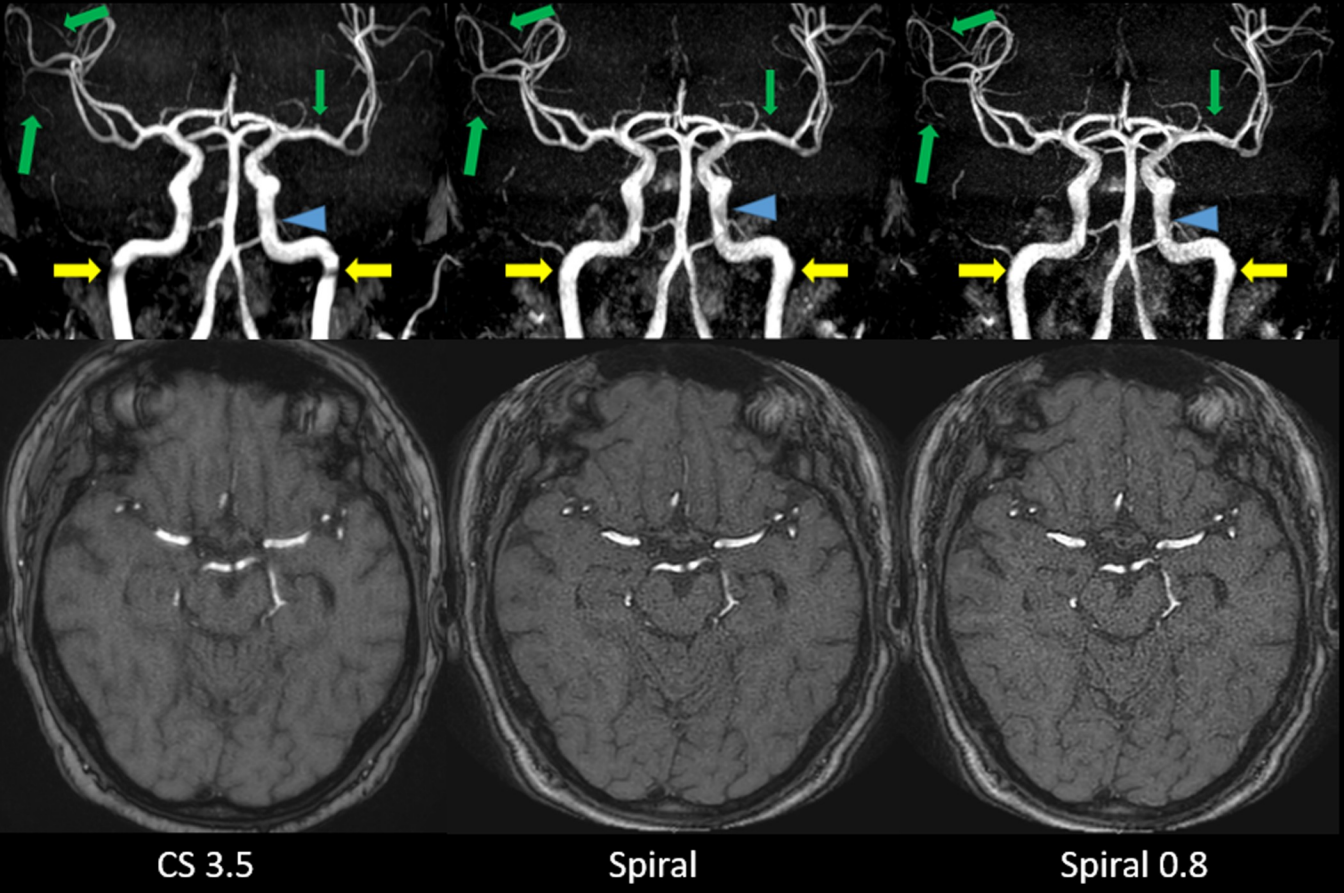
Coronal whole volume MIP and source images of CS 3.5 (A), Spiral (B) and Spiral 0.8 (C). Artifacts at the turn of the vertical to the horizontal petrous segment of both internal carotid arteries are reduced on both Spiral-TOFs compared to the CS 3.5 (yellow arrows). However, slightly more inhomogeneous central signal in the cavernous segment of the left internal carotid artery (blue arrowheads) can be seen on both Spiral-TOFs compared to the more homogeneous central signal on the CS 3.5 TOF. As for peripheral vessels, inconsistent depiction of small arterial branches arising from the M2 and M3 Segments of the right middle cerebral artery and from the M1 segment of the left middle cerebral artery can be seen on the CS 3.5 compared to consistent depiction on both Spiral-TOFs (green arrows).

**Fig 3:**
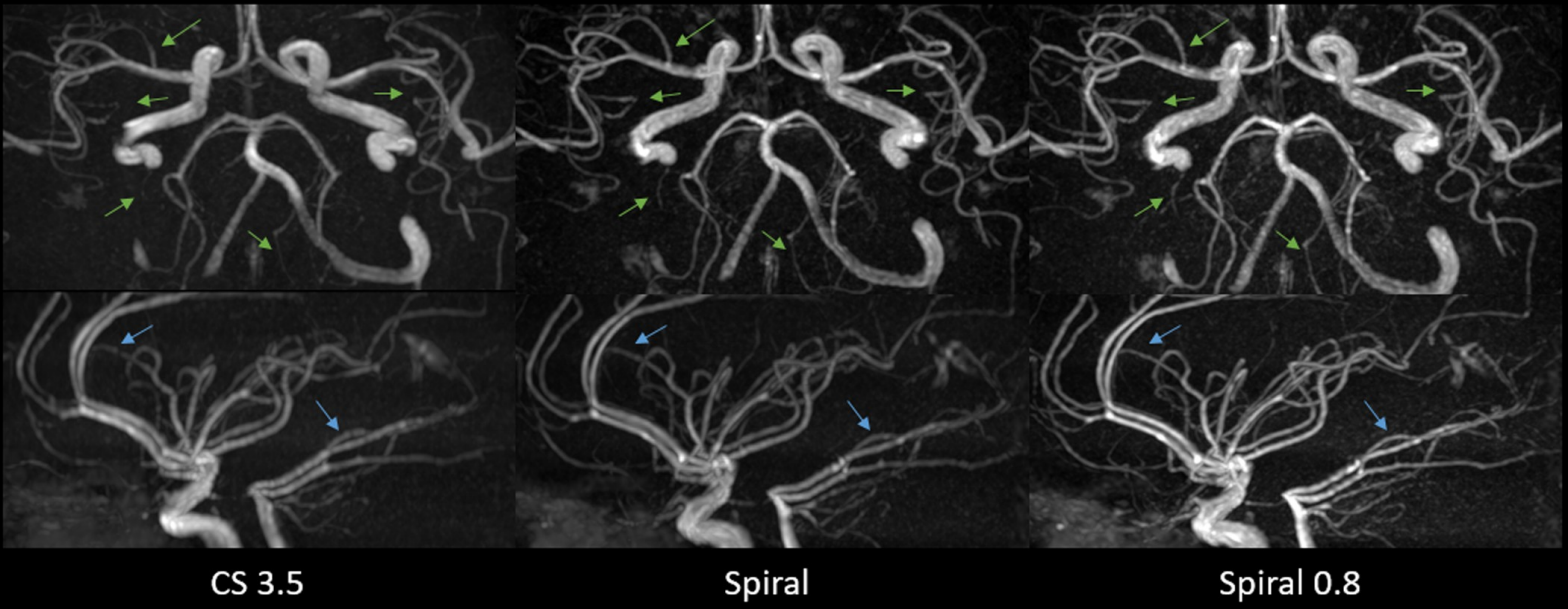
Axial and sagittal MIP images showing improved peripheral image quality on Spiral and Spiral 0.8 images as compared to CS 3.5 images. The green and blue arrows mark small vessels that do not appear on CS 3.5 images but are visible on Spiral and Spiral 0.8 images. Furthermore, an improved visualization of small peripheral vessels on Spiral and Spiral 0.8 images can be seen. Images were deliberately adjusted in terms of vessel hyperintensity to make differences in small vessel visualization more apparent.

For both readers there were no significant differences between the CS 3.5, Spiral and Spiral 0.8 sequences in terms of central image quality (p = 0.568, p = 0.954). However, both readers found the Spiral and Spiral 0.8 sequences to outperform the CS 3.5 sequence in terms of peripheral image quality (p<0.001, p<0.001, p<0.001, p<0.001). Upon questioning and reinspection of relevant cases, the readers noted that they appreciated the enhanced peripheral visibility of small vessels on Spiral images. Concerning central image quality they noticed that in direct comparison, the Spiral MIP images appeared with less effective background suppression in the central regions around the internal carotid arteries compared to the CS 3.5 sequence, especially when the patient appeared to have more subcutaneous fat tissue in the facial region. In such cases, the vessels on MIPs seemed slightly less hyperintense compared to the background. This effect was however not observed on source images (which could be confirmed by CR measurements). Furthermore, by optimizing the windowing on Spiral MIP images the issue could be virtually resolved. This may also explain, why nonetheless no significant difference in central image quality was observed. An overview of the data is given in **Table 2**and visualized in **Fig 1**. Representative imaging examples are provided as **Figs 2 and 3**.

**Table 2:** Overview of the data from qualitative image quality assessment.

	Reader 1 Central Image Quality (median; [Interquartile Range IQR])	Reader 1 Peripheral Image Quality (median; [Interquartile Range IQR])	Reader 2 Central Image Quality (median; [Interquartile Range IQR])	Reader 2 Peripheral Image Quality (median; [Interquartile Range IQR])
CS 3.5	4; [4, 5]	4; [3.75, 4]	4; [4, 5]	4; [3, 4]
Spiral	4; [3.75, 5]	5; [4, 5]	4; [4, 5]	4; [4, 5]
Spiral 0.8	4; [3, 5]	5; [4.75, 5]	4; [4, 5]	5; [4, 5]

### Lesion and variation assessment

**Fig 4:**
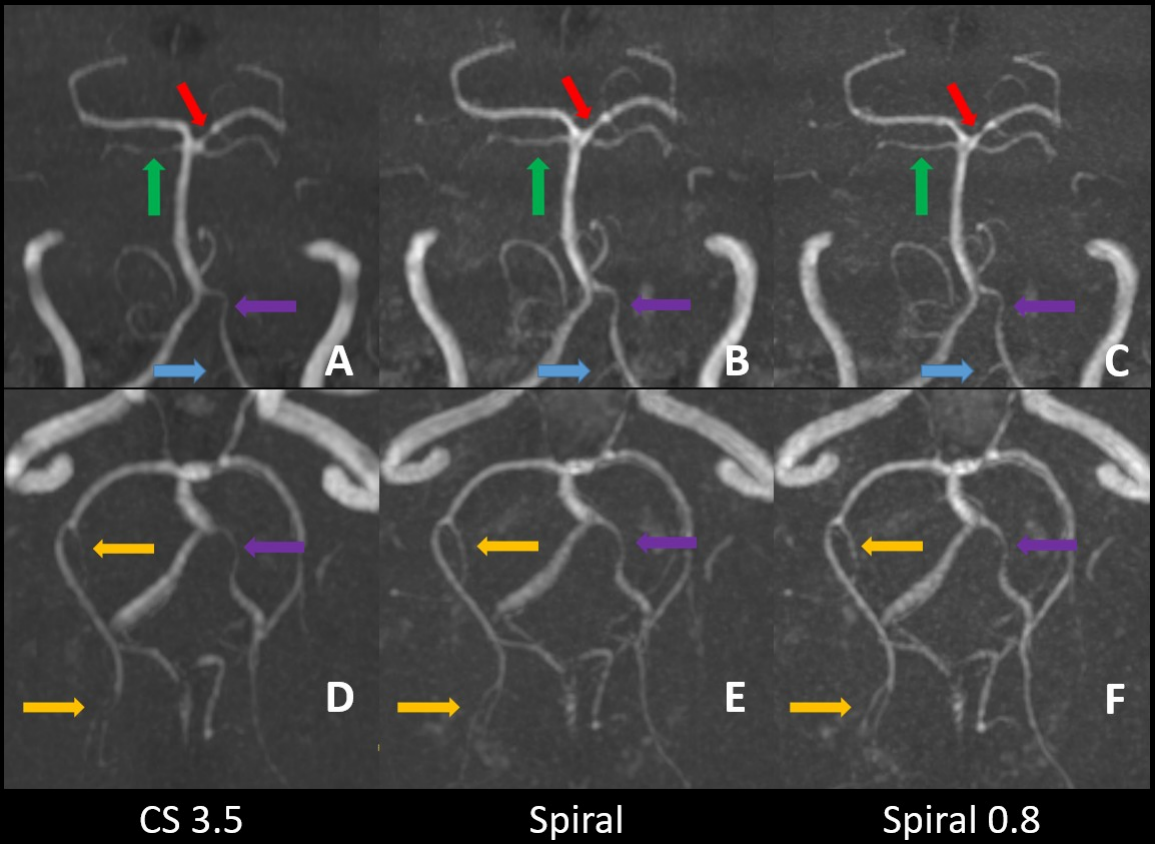
**Zoomed coronal and transverse MIP images of CS 3.5 (A,D), Spiral (B,E) and Spiral 0.8 (C,F).** A moderate stenosis in the P1 segment of the left posterior cerebral artery (red arrows) is well depicted in all three sequences. Small arterial branches as the right superior cerebellar artery (green arrows), the left posterior inferior cerebellar artery (blue arrows) and arterial branches of the right posterior cerebral artery arising from the P2 to P4 segments (yellow arrows) are more consistently shown on Spiral-TOFs than on CS 3.5 TOF. Hypoplastic arterial segments as the distal V4 segment of the vertebral artery on the left side are visible on all three sequences (purple arrows).

**Fig 5:**
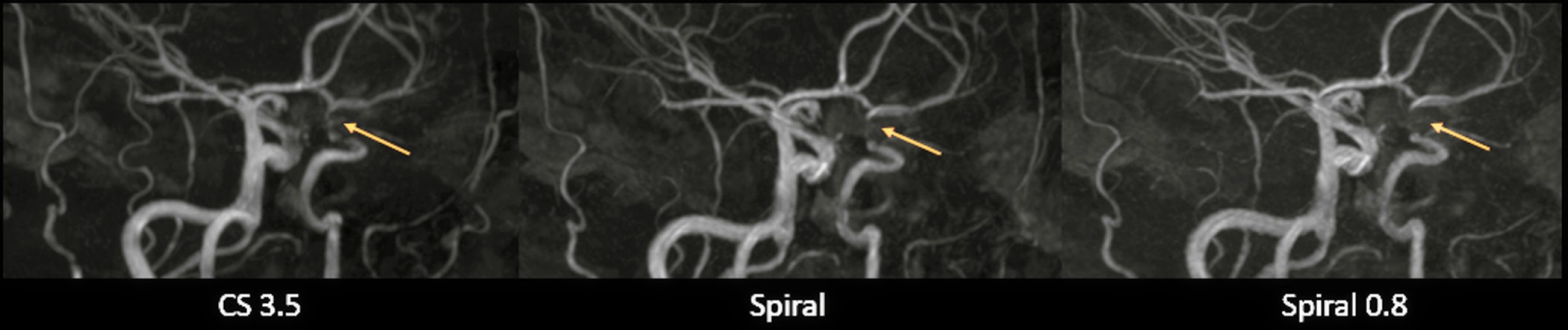
MIP images showing supraclinoid internal carotid artery occlusion on all three sequences with slightly improved visualization on Spiral and Spiral 0.8 images.

**Fig 6:**
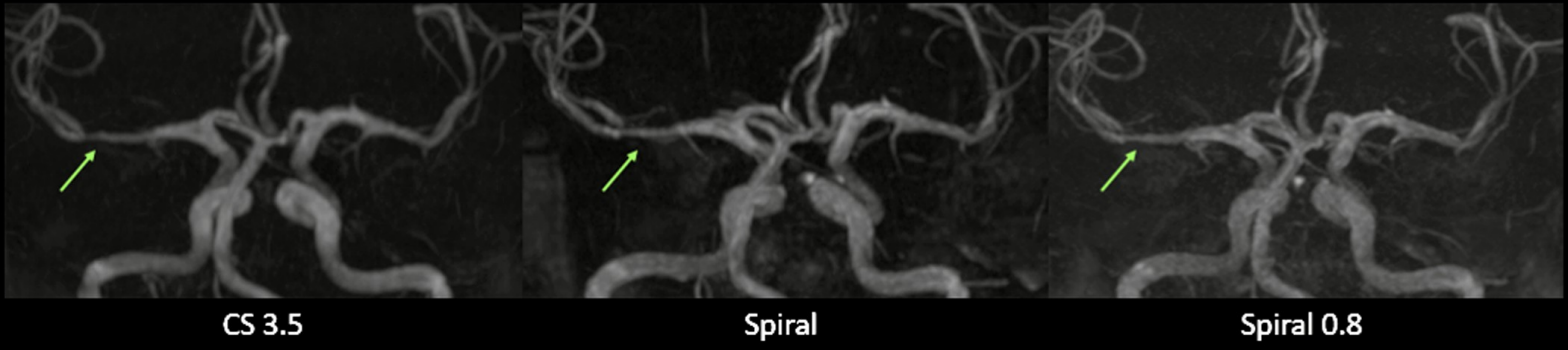
MIP images showing a tubular stenosis of the distal M1 segment of the middle cerebral artery.

**Fig 7:**
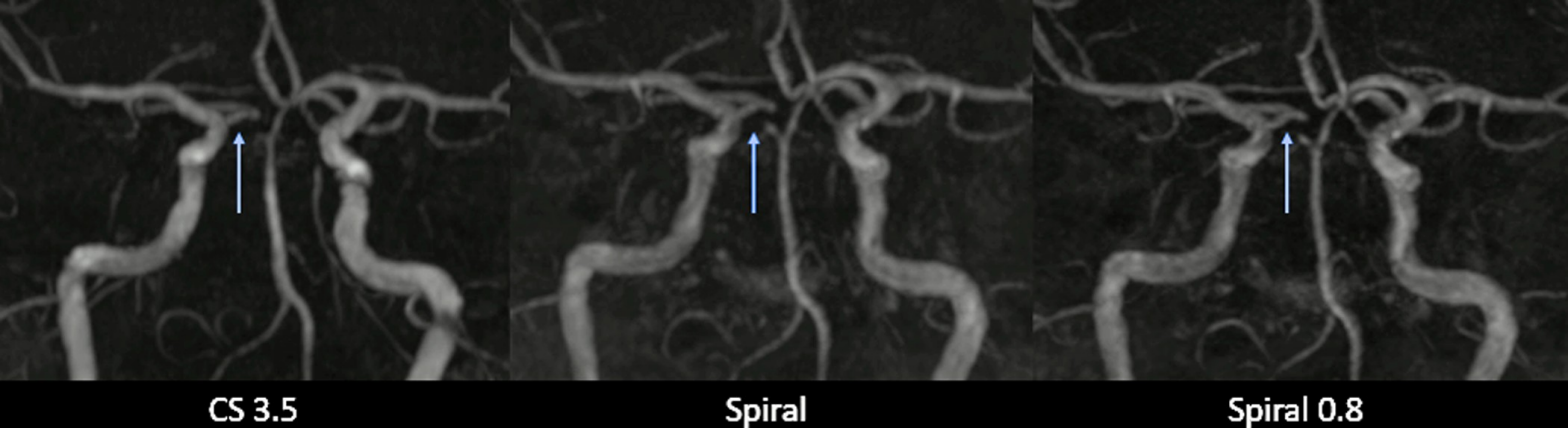
MIP images showing an embryonic posterior cerebral artery with missing P1 segment of the posterior cerebral artery on the right side.

Forty-two patients presented with at least one pathologic finding such as stenosis or an anatomical variant. An overview of the data is given in **Table 3**. In brief, interstudy agreement between the three TOFs ranged from good (*W* = 0.638, p<0.001) to excellent (*W* = 1, p<0.001) for reader 1 and from good (*W* = 0.67, p<0.001) to excellent (*W* = 1, p<0.001) for reader 2. Thus the three sequences presented with a comparable diagnostic efficacy. Interestingly reader 2 diagnosed more stenoses than reader 1, which may be attributable to differences in clinical experience (7 years vs 30 years). Furthermore, while not significant, there were some notable differences in the scoring of the category "decreased branches" between the three TOFs for both readers. Upon questioning and reinspection of some relevant cases, the readers concluded that decreased branches were mostly diagnosed on small peripheral vessels. As the Spiral-TOFs enable better peripheral image quality and vessel visualization, vessels were often visible on Spiral-TOF images but not or only partially on CS 3.5 images thus leading to the misdiagnosis of decreased branches on CS 3.5 images. Representative imaging examples of pathologic findings are provided in **Figs 4–7**.

**Table 3:** Overview of the data from lesion and variation assessment.

	Reader 1 CS 3.5	Reader 1 Spiral	Reader 1 Spiral 0.8	Reader 1 Kendall W Interstudy agreement (*W*, p-value)	Reader 2 CS 3.5	Reader 2 Spiral	Reader 2 Spiral 0.8	Reader 2 Kendall W Interstudy agreement (*W*, p-value)
Mild Stenosis	31	26	25	0.74, p<0.001	37	34	30	0.745, p<0.001
Moderate Stenosis	16	21	20	0.638, p<0.001	22	19	20	0.678, p<0.001
Severe Stenosis	12	12	12	0.779, p<0.001	13	19	19	0.782, p<0.001
All Stenosis	59	59	57	0.876, p<0.001	72	72	69	0.909, p<0.001
Occlusion	2	2	2	1, p<0.001	2	2	2	1, p<0.001
Aneurysm	2	2	2	1, p<0.001	2	2	2	1, p<0.001
Decreased Branches	13	10	5	0.842, p<0.001	16	11	5	0.805, p<0.001
Congenital Hypoplasia	63	63	63	1, p<0.001	63	63	63	1, p<0.001
Congenital Aplasia	9	9	9	1, p<0.001	9	9	9	1, p<0.001
Congenital Variant Branching	24	24	24	1, p<0.001	24	25	25	0.986, p<0.001

### Interreader agreement

The interreader agreement between reader 1 (S.S) and 2 (A.S) for image quality and lesion and variation assessment ranged from good (*K* = 0.638) to excellent (*K* = 1). As expected, interreader agreement for stenoses ratings were lower than in other categories due to the differences between reader 1 and 2. An overview is given in **Table 4**.

**Table 4:** Overview of the interreader agreement from qualitative image quality and lesion and variation assessment.

Interreader Agreement	CS 3.5 weighted Cohen's Kappa (*K*); [95% confidence interval]	Spiral weighted Cohen's Kappa (*K*); [95% confidence interval]	Spiral 0.8 weighted Cohen's Kappa (*K*); [95% confidence interval]
Image Quality Central	0.903; [0.791, 1.000]	0.899; [0.803, 0.994]	0.849; [0.735, 0.964]
Image Quality Peripheral	0.925; [0.819, 1.000]	0.675; [0.461, 0.889]	0.827; [0.649, 1.000]
Mild Stenosis	0.757; [0.585, 0.930]	0.725; [0.560, 0.890]	0.792; [0.635, 0.950]
Moderate Stenosis	0.638; [0.390, 0.885]	0.797; [0.618, 0.977]	0.868; [0.715, 1.000]
Severe Stenosis	0.639; [0.388, 0.891]	0.743; [0.563, 0.922]	0.743; [0.563, 0.922]
All Stenosis	0.763; [0.614, 0.912]	0.705; [0.542, 0.868]	0.704; [0.539, 0.868]
Occlusion	1.000; [1.000, 1.000]	1.000; [1.000, 1.000]	1.000; [1.000, 1.000]
Aneurysm	1.000; [1.000, 1.000]	1.000; [1.000, 1.000]	1.000; [1.000, 1.000]
Decreased Branches	0.784; [0.607, 0.961]	0.944; [0.853, 1.000]	1.000; [1.000, 1.000]
Congenital Hypoplasia	1.000; [1.000, 1.000]	1.000; [1.000, 1.000]	1.000; [1.000, 1.000]
Congenital Aplasia	1.000; [1.000, 1.000]	1.000; [1.000, 1.000]	1.000; [1.000, 1.000]
Congenital Variant Branching	1.000; [1.000, 1.000]	0.97; [0.908, 1.000]	0.97; [0.908, 1.000]

### Quantitative image quality—contrast ratio (CR)

There were no significant differences between the CS 3.5 (0.47 ± 0.05), Spiral (0.48 ± 0.06) and Spiral 0.8 (0.48 ± 0.06) sequences in terms of CR (p = 0.534). Upon questioning and reinspection of some relevant cases the readers noted that while vessels were depicted equally well on all source images (in terms of brightness and delineation in comparison to the background as quantified by CR) the background seemed slightly noisier on Spiral source images (i.e. reduced signal to noise ratio). This effect was most pronounced on the Spiral 0.8 images, which may be a tradeoff of the higher resolution [8]. The data is presented visually in **Fig 1**.

## Discussion

In this intra-individual comparison study, we investigated the clinical feasibility of Spiral-TOF imaging at 1.5T and showed that two Spiral-TOFs exhibited comparable diagnostic efficacy and equal or better qualitative and quantitative image quality as a CS accelerated TOF (CS 3.5). Most importantly, the Spiral sequence achieved an acquisition time of below 2 min (01:32 min) thus representing a 50.54% reduction in scan time compared to the CS 3.5.

While spiral readout techniques have previously been employed for other imaging modalities such as coronary artery imaging or postcontrast brain imaging [14–18] it has only recently become a viable option for TOF imaging [8,9].

Sartoretti et al. investigated the use of Spiral-TOFs for intracranial vessel imaging at 1.5T in an in vitro setting and in healthy volunteers. Spiral-TOFs were found to achieve improved qualitative and quantitative image quality compared to PI and CS accelerated TOFs and were able to accurately depict the dimensions of vessels [8].

In their pilot study, Greve et al. examined the use of a Spiral-TOF sequence in a clinical setting at 3T. Their Spiral-TOF exhibited a comparable diagnostic efficacy as their CS accelerated TOF and enabled improved small vessel visualization. The authors thus concluded that Spiral-TOF imaging was clinically feasible at 3T. However, they described some drawbacks of the Spiral-TOF for the visualization of intraosseous and intradural segments of the internal carotid artery [9].

In our study we also showed that clinical Spiral-TOF imaging may be feasible at 1.5T. However, in contrast to Greve et al. we did not observe reduced image quality of the intraosseous and intradural segments of the internal carotid artery on our Spiral-TOF images compared to our CS 3.5 images. While we did not assess the image quality of these segments separately, we evaluated these segments as part of our rating category "central image quality" and found no significant difference between the three TOFs. However, while not significant, slight differences in background suppression between the CS 3.5 and Spiral MIP images were observed that could be virtually resolved by optimising the windowing. We hypothesize that the previously described loss of image quality at the skull base [9] may be influenced by the field strengths of the scanner (3T vs 1.5T). For example, B_0_ field inhomogeneity may be more prominent at 3T thus resulting in a loss of image quality.

As for the two Spiral-TOFs used in the current study, it has previously been hypothesized that the isotropic Spiral 0.8 sequence may be more suitable for clinical grade imaging compared to the Spiral sequence [8]. However in this study the Spiral 0.8 did not offer substantial advantages over the Spiral sequence. Yet for the visualization of very small and peripheral vessels, the Spiral 0.8 may still prove to be advantageous due to its higher resolution. This is supported by the fact that the Spiral 0.8 sequence achieved the highest scores of all three TOFs in the category peripheral image quality and this may have also influenced the differences observed under the category decreased branches in lesion and variation assessment.

Lastly, the value of Spiral-TOF imaging in terms of reducing acquisition times has to be strongly emphasized. The Spiral sequence achieved a reduction of slightly over 50% compared to the CS 3.5 TOF while the isotropic Spiral 0.8 achieved a reduction of 29%. Thus, in case of the Spiral sequence, high quality intracranial vessel imaging could be achieved at unprecedented scan times ranging below 2 min [8]. Firstly, this may be desirable for MR institutions serving a large number of patients and secondly for patients in pain or discomfort which may greatly appreciate reductions in procedure times [13].

For the future, the acquisition time may possibly be shortened even further by combining compressed sensing [23, 25–29] and parallel imaging technology together with spiral acquisition methods. However, the feasibility of such a combination is unknown and would have to be tested.

Certain limitations of this study should be acknowledged: Firstly, the sample size was quite small yet comparable to that of other studies assessing the clinical feasibility of sequences [1,19,21,22, 30,31]. Specifically, due to the limited sample size, certain pathologies only appeared very rarely (i.e. aneurysms) and thus our ability in demonstrating comparability between the three TOFs may have been limited [1]. Secondly, we are aware of the numerous formulas used to determine CR and acknowledge that results may differ when applying other formulas. Thirdly, while in line with other similar studies [1], we did not use DSA as a gold standard for lesion and variation assessment. Lastly, a relatively heterogeneous group of patients was included in the study for assessment of diagnostic performance [1].

## Conclusion

To conclude, we provide data on the clinical performance of two Spiral-TOFs in comparison with a CS accelerated TOF (named CS 3.5) and show that Spiral-TOFs deliver high quality intracranial vessel imaging at 1.5T in a clinical setting at unprecedented scan times. Spiral-TOFs may thus become a viable option for clinical intracranial vessel imaging.

## Supporting information

S1 File Data. The file "Data" contains all relevant data used in this study. (XLSX)

## Abbreviation key

CS: Compressed Sensing
PI: Parallel Imaging
TFE: Turbo Field Echo
AcoA: Anterior Communicating Artery
PcoA: Posterior Communicating Artery
CR: Contrast Ratio
SI: Signal Intensity
